# Emotional Intelligence in Elementary School Children. EMOCINE, a Novel Assessment Test Based on the Interpretation of Cinema Scenes

**DOI:** 10.3389/fpsyg.2019.01882

**Published:** 2019-08-14

**Authors:** Santiago Sastre, Teresa Artola, Jesús M. Alvarado

**Affiliations:** ^1^University Center Villanueva, Complutense University of Madrid, Madrid, Spain; ^2^Department of Psychobiology & Behavioral Sciences Methods, Faculty of Psychology, Complutense University of Madrid, Madrid, Spain

**Keywords:** emotional intelligence, elementary children, test validity, films, emotional problems and disorders

## Abstract

The aim of the present study was to validate a new procedure, called EMOCINE test, to measure the perception and understanding branches of emotional intelligence (EI) in 1,448 children, aged between 8 and 13 years, from elementary schools. This new tool consists of watching 15 cartoon film clips and interpreting them according to children’s own emotional perception and comprehension. After completing the test, the participants were classifie, according to their responses and by using the latent class analysis, as naïve (16.1%), over-interpretative (33.5%), and sensitive (50.4%). An increase in sensitive responses was observed depending on participants sex (girls higher than boys), and academic grade (increasing over the grades). Participants from the sensitive latent class had a significant better behavior in the school/classroom, compared with those from naïve and over-interpretative groups. Likewise, significant differences between latent classes were observed in many psychological, emotional, and behavioral problems (mainly between sensitive vs. insensitive by naivety) such as: depression, post-traumatic symptomatology, attention problems, aggression, family problems, problems at school, problems with classmates, integration and social competence. In conclusion, EMOCINE is a novel and promising tool for the measurement of the perception and comprehension components of EI. The test, employing film clips, is more realistic and motivating than others using static images or written descriptions. Moreover, the test can predict or identify emotional and behavior problems in children.

## Introduction

Emotional intelligence (EI) is a concept originally introduced by Salovey and Mayer (1990) in and refers to four branches or interrelated factors, i.e., the ability for perceiving, understanding, managing, and evaluating emotions (Salovey and Mayer, 1990; Mayer and Salovey, 1997; Mayer et al., 2016). For these authors, EI is considered as a member of the class of broad intelligences focused on hot information processing. Mayer et al. (2016, p. 292) differentiated between “cool intelligences, which deal with impersonal knowledge, such as verbal intelligence and math abilities,” and “hot intelligences which involve reasoning with information of significance to the individual,” such as social acceptance, identity coherence, and emotional well-being. EI, social intelligence, and personal intelligence fall within this category. These three types of intelligences should therefore be closely related (Mayer et al., 2016). There is a consensus about the importance of emphasizing the acquisition of emotional skills in all educational levels, showing that emotional consciousness is associated with highly positive consequences, improving interpersonal relationships, group cohesion, and emotional well-being (Brackett et al., 2006; Martins et al., 2010; Fernández-Berrocal and Extremera, 2016; García-Sancho et al., 2017; Fotopoulou et al., 2019). This evidence has motivated the development of different programs for enhancing emotional abilities, and the creation of novel tools for its measurement.

Depending on whether the objective of the measurement is perception of one’s emotional functioning or ability in emotion processing, a number of different instruments have been created; self-report questionnaires (frequently short questions, answers using Likert scales) offer distinct advantages, such as the ease of application and correction (Conte, 2005; Goldenberg et al., 2006). However, people are often poor at estimating their own level of intelligence, both general intelligence or EI (Brackett et al., 2006). Self-reports provide a subjective evaluation of the own perceived emotional capacity that, in some cases, can be biased (Vazire, 2010). Responses in self-questionnaires are often influenced by mood, social desirability and self-esteem of the participants. This limitation is especially relevant in adolescents or children who have a poor capacity of introspection and therefore are not effective for accurately determining their own-emotional skills. By contrast, ability tests are more objective and avoid subjectivity and overlapping with other personal traits. A number of ability tests for evaluating EI in adults have been published in recent years (Mayer et al., 2002; Barraca et al., 2009; Śmieja et al., 2014; Sastre et al., 2018). The Mayer-Salovey-Caruso Emotional Intelligence Test (MSCEIT) is the most representative test from this group (Mayer et al., 2002) that has been designed to measure the four branches of the Mayer and Salovey model (Mayer and Salovey, 1997).

Barraca et al. (2009) developed a new procedure to evaluate IE ability, focused on perception and understanding branches (Sensitivity to Social Interactions Test, TESIS). TESIS consisted of watching 15 film clips with emotional situations and then choosing between 5 answers to questions developed to each clip. The test was aimed at differentiating individuals (adults) emotionally sensitive from those emotionally insensitive. Subsequently, Sastre et al. (2018) adapted TESIS to an adolescent population (named TESIS-SEC) and validated it with 1,536 students from first grades of secondary school. Results revealed a suitable validity and a good fit with TESIS (Barraca et al., 2009). Nevertheless, given the complexity and content of the film clips, TESIS-SEC cannot be used in children, therefore we designed a new test: EMOCINE (EMOtion in the CINEma-scenes), similar to TESIS. EMOCINE substitutes the narration of situations used in MSCEIT (Mayer et al., 2002) and MSCEIT-YV for early adolescents (Rivers et al., 2012), by the viewing of film scenes, and obtains a measure that does not depend on verbal comprehension.

The aim of this research was to perform the first validation of EMOCINE. To this end, validity of the content, validation of construct, and the relation with other relevant variables were obtained. Taking into account the available evidence about other measurements of the construct, it was hypothesized that the frequency of psycho-emotional problems would be lower in children with higher EI (i.e., Fernández-Berrocal et al., 2018). Moreover, since EI, as we have already stated, is considered as a broad hot intelligence (Mayer et al., 2016), comparisons with other “hot” intelligences such as social intelligence should be expected, such as a better perception and acceptance by the group in children with higher EI (Zavala-Berbena et al., 2008; Fotopoulou et al., 2019). Regarding sex and age, it would be expected that girls achieve higher scores than boys, in agreement with studies carried out done with teenagers (Rivers et al., 2012).

## Materials and Methods

### Participants

A total of 10 public and private schools, from 8 Spanish Autonomous Communities (regions), were selected and agreed to participate in the study. The number of participants was 1,448 (48.9% girls, and 51.0% boys), aged between 8 and 13 years, and studying from third to sixth grade in elementary school. The principal of each school informed their parents about the study and objectives. All parents signed an informed consent for the participation of the children. This study was approved by the ethics committee of the Faculty of Psychology of the Complutense University of Madrid: “Comisión Deontológica de la Facultad de Psicología de la Universidad Complutense de Madrid (Ref. 2018/19-013).”

### Instrument

The EMOCINE test consists of watching 15 clips or scenes from cartoon movies and interpreting them according to the emotional perception and comprehension of the situation. After viewing each film clip, a voice-over raises a question, and participants have to choose between 3 answers (shown in an answer sheet). One of the answers is considered as sensitive, another as insensitive due to naivety, and another as insensitive due to over-interpretation.

Several previous studies were carried out to achieve the final version of EMOCINE. First, a panel of five experts worked on the identification and selection of the adequate cartoon film clips (no longer than 2 min each, from Disney movies) that expressed implicit emotional situations that were not perceived by all children. Adequation and difficulty of items were evaluated in 71 students from elementary school. Children showed a favorable response to the test. Next, pertinent questions about the emotional perception and understanding of each scene were developed by the same experts. Then, open answers to those questions were collected from a cohort of 384 children. A qualitative analysis was performed with the 7,680 open answers obtained about the 20 film scenes. All these answers were analyzed and classified as sensitive, insensitive due to naivety, or insensitive due to over-interpretation, considering as sensitive those responses that indicated that the child perceived accurately what was happening in the scene and understood the emotions it reflected. Responses were classified as naïve when they were not related to the emotions shown, or/and when they were merely descriptive. Responses were classified as over-interpretive when they were more than a mere description, but the interpretation of the emotional meaning was not correct, or when they included additional information that was not in the scene and that could not be deduced from the film. The next step consisted of selecting the three most frequent answers in children from each category. Once the test was designed, a pilot study (20 scenes, and 3 answers) was applied to 134 children. Five film clips showed low discrimination rates and were then discarded out from the final version of EMOCINE (15 scenes and 3 answers). The present study was carried out by using this final version of EMOCINE. Film clips and questions are described in Supplementary Table S1.

### Studied Variables

Differences in the results obtained by the three latent groups in their scores in emotional perception and understanding were evaluated in relation to the following variables: sex; age; academic performance; general behavior in school/classroom; social relationships and sociometric status; and emotional and behavioral problems. Tutors determined the general behavior of each student in the school/classroom by a five-item Likert scale, ranging between 1 (very bad behavior), 2 (bad behavior), 3 (normal behavior), 4 (good behavior), and 5 (very good behavior). Social relationships and social status of students were evaluated by using the SOCIOMET test, consisting of four questions that students had to answer (González and García, 2010). The questions were as follows: (1) Who are the three classmates that you choose as best friends? List them from more- to less- best friends; (2) Who are the three classmates that you do not like as friends. List them from less to more; (3) Who are the three classmates that you think have chosen you as a best friend? List them from more to less, and (4) Who are the three classmates that you think have chosen you as someone they don’t like? List them from less to more. Based on this information, we calculated the total number of times that a student was chosen as best friend, rejected as a friend, thought he/she was chosen as a best friend, and thought he/she was rejected as a friend. The detection of emotional and behavior problems in students was carried out by completing the SENA (*Sistema de Evaluación de Niños y Adolescentes* – Evaluation system for children and adolescents) (Fernández-Pinto et al., 2015). This self-report consists of 134 items providing information about 22 scales/indexes: depression, anxiety, social anxiety, somatic complaints, post-traumatic symptomatology, attention problems, hyperactivity-impulsivity, problems with the control of anger, aggression, challenging behavior, family problems, problems with school, problems with classmates, problems of emotional regulation, self-esteem, integration and social competence, index of emotional problems, index of behavior problems, index of problems in executive functions, index of contextual problems, index of personal resources, and global index of problems. The response to each item was Yes or No. The SENA self-report was completed by paper-and-pencil, in approximately 30–40 min. The reliability of SENA was evaluated in a wide validation study with 2,250 participants aged between 3 and 18 years old. Reliability was above 0.7 on almost all scales. Results showed adequate evidences of the validity of the construct (Sánchez-Sánchez et al., 2016). All tests were completed during two class sessions.

### Statistical Analysis

The assignation of participants into a corresponding group/class (sensitive, over-interpretative, or naïve) was performed by using a latent class analysis (LCA), available with R version 3.2.1 (package poLCA) (Linzer and Lewis, 2011).

Categorical variables were expressed as relative and absolute frequencies, whereas continuous ones as mean and standard deviation (SD). Comparisons of latent classes regarding sex and academic grade in elementary school were performed with Pearson’s chi-square test. Comparisons of latent classes regarding academic performance, general behavior in school/classroom, sociogram, and emotional and behavioral problems evaluated through the SENA questionnaire were carried out by using a multiple analysis of the variance (ANOVA). Statistical significance was established with *p* < 0.05. All statistical analyses were carried out by using SPSS 22.0 version.

## Results

**FIGURE 1:**
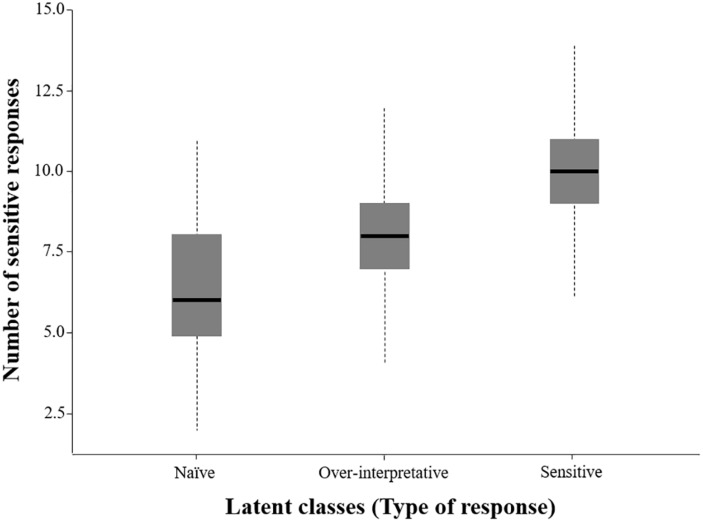
Distribution of latent classes in the population of children from elementary school.

Participants from the present study were then assigned into the latent class that provided the highest probability of assignment (Linzer and Lewis, 2011). An example of LCA syntaxis with simulated data (aimed at facilitating the comprehension of latent class assignment) is included as Appendix I. A total of 233 (16.1%), 485 (33.5%), and 730 participants (50.4%) constituted the naïve, over-interpretative, and sensitive latent classes after completing EMOCINE test, respectively. The distribution of sensitive responses according to latent classes is shown in Figure 1 (see univariate distribution for all responses in the Supplementary Material).

The factorial confirmatory analysis with robust weighted least squares estimation showed good fit to the unidimensional model for sensitive responses: χ^2^ (90) = 133.35, *p* < 0.01; Root Mean Square Error of Approximation (RMSEA) = 0.018 (0.011; 0.025); Root Mean Square Residual (SRMR) = 0.049; Comparative Fit Index (CFI) = 0.97; Non-Normed Fit Index (NNFI) = 0.97; Standardized Root Mean Square Residual (SRMR) = 0.049. Marginal reliabity (based on the average conditional standard errors) = 0.66.

**FIGURE 2:**
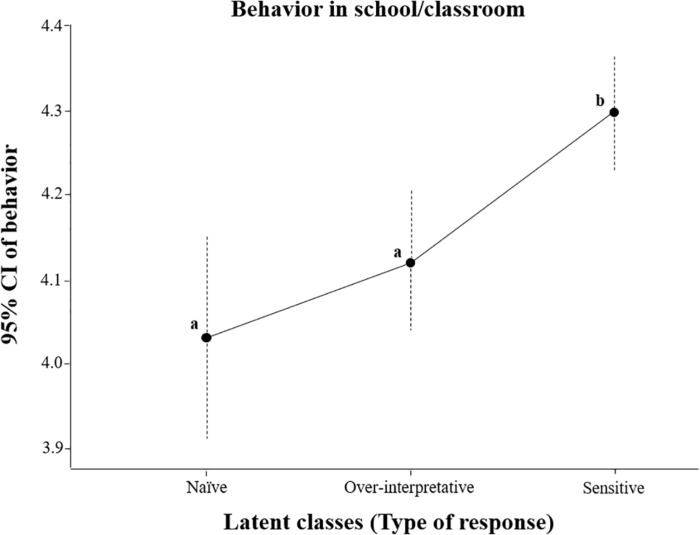
Distribution of latent classes regarding behavior in school/classroom. Different letters (a,b) indicate significant differences in sensitive responses.

An increase in sensitive responses was observed depending on the sex of participants (girls higher than boys, 433 vs. 299; χ^2^(2) = 41.38, *p* < 0.001), and academic grade in elementary school (increasing over the years, 107 sensitive responses in the third grade, 165 in the second, 204 in the fifth, and 256 in the sixth; χ^2^(6) = 151.08, *p* < 0.001). The number of participants constituting the naïve and over-interpretative latent classes significantly decreased over academic grades. The analysis of variance shows that participants from the sensitive group had a significant better behavior [*F*(2,1114) = 9.07, *p* < 0.001, ηp2 = 0.02] in the school/classroom compared with those from naïve and over-interpretative groups (Figure 2).

**FIGURE 3:**
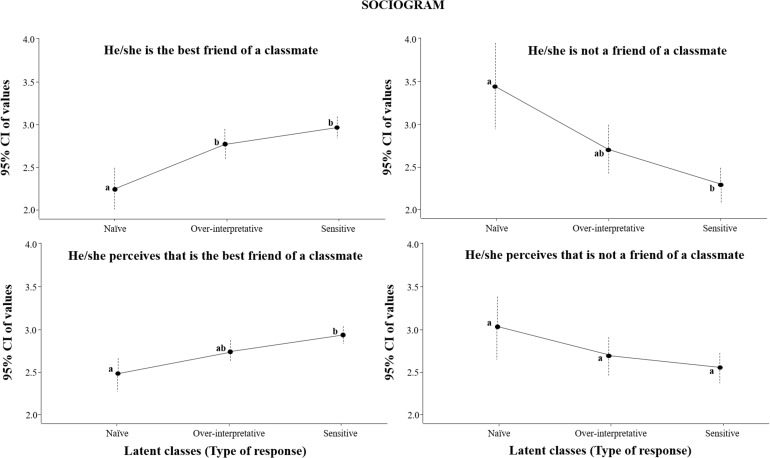
Sociogram results regarding latent classes. Different letters (a,b) indicate significant differences in sensitive responses.

A significant [*F*(2,1445) = 12.29, *p* < 0.001, ηp2 = 0.02] higher number of children from the sensitive group were considered the best friend of a classmate and they were aware of that (Figure 3), compared with those from the naïve group. Furthermore, a significant [*F*(2,1445) = 11.25, *p* < 0.001, ηp2 = 0.02] less number of participants from the sensitive group were not considered as a desired friend, and they perceived it so [*F*(2,1445) = 9.62, *p* < 0.001; ηp2 = 0.01; *F*(2,1445) = 2.68, *p* = 0.069, ηp2 < 0.01].

**FIGURE 4:**
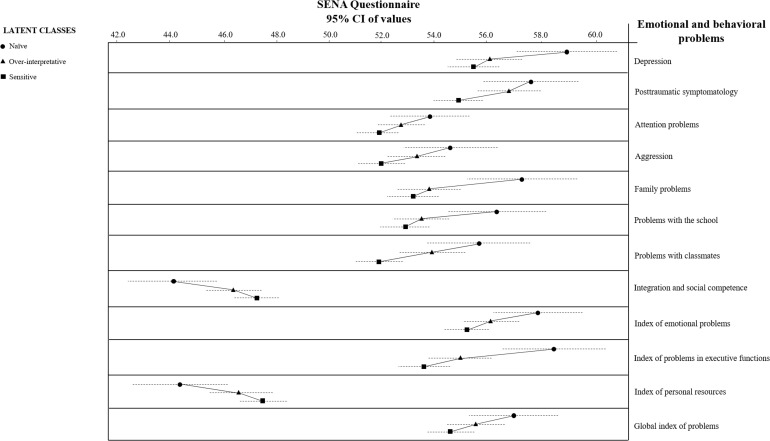
Emotional and behavioral problems from SENA questionnaire showing significant differences regarding latent classes.

Multivariate analysis of variance shows that emotional and behavioral problems evaluated by the SENA questionnaire showing significant differences (Wilks’s lambda = 0.90, *p* < 0.001, ηp2 = 0.05) regarding latent classes (mainly between sensitive vs. insensitive by naivety) were as follows (Figure 4): depression (*p* = 0.006), post-traumatic symptomatology (*p* = 0.009), attention problems (*p* = 0.030), aggression (*p* = 0.041), family problems (*p* = 0.001), problems with the school (*p* = 0.003), problems with classmates (*p* < 0.001), integration and social competence (*p* = 0.003), index of emotional problems (*p* = 0.020), index of contextual problems (*p* < 0.001), index of personal resources (*p* = 0.006), and global index of problems (*p* = 0.036).

## Discussion

The evaluation of emotional perception and emotional comprehension in children by using cartoon film clips (EMOCINE) has provided first promising evidences of validity. Significant differences have been found between latent classes regarding sex and academic grade. Women frequently are better at perceiving, understanding, and describing their own emotions and those of others (Barrett and Bliss-Moreau, 2009; Fischer et al., 2018). Sex differences in EI have been strongly demonstrated with ability tests, showing that women obtain higher scores than men (Naghavi and Redzuan, 2011; Rivers et al., 2012; Śmieja et al., 2014). By contrast, studies using self-report questionnaires have shown non-conclusive results (Mayer et al., 1999; Van Rooy et al., 2005; Brown and Schutte, 2006; Fischer et al., 2018). Perception and understanding of emotional situations in adolescents, measured with TESIS-SEC, was also significantly associated with sex and interpersonal intelligence (Sastre et al., 2018). Although a matter of debate (Webb et al., 2013; Sharma, 2017), it has been proposed that EI increases with age, because of the development of EI ability (Rivers et al., 2012), or by the accumulation of knowledge (Burns et al., 2007). Our results indicated that the sensitive latent class increased over the academic grade (from third to sixth) in elementary school. Furthermore, a lower emotional sensitivity has been associated with maladaptive behavior (drug and alcohol consumption, and fighting) (Brackett et al., 2004; Omori et al., 2006). In children, a higher emotional sensitivity has been positively correlated with leadership, adaptability, and study skills (Brackett and Katulak, 2006). The validity of EMOCINE in relation with other relevant variables was therefore confirmed by its correlation with emotional and behavior problems detected with SENA questionnaire. Based on the results of this self-report, the children from the sensitive group had a significant lower risk for depression, post-traumatic symptomatology, attention problems, aggression, family problems, problems with the school, problems with classmates, compared with those from the insensitive by naivety group. In fact, sensitive individuals showed significant lower indexes of emotional, contextual, and global problems. These results are in agreement with previous studies with Spanish adolescents, showing a positive relationship between EI ability and personal and scholar adjustment (Fernández-Berrocal et al., 2018). These results are also in concordance with previous studies, in which emotional sensitivity is negatively associated with hyperactivity, anxiety, attention and learning problems, behavior problems, and aggression (Brackett and Katulak, 2006). Additionally, our results provide evidence that individuals with high EI have higher probability for integration and better social acceptance by their peers as previous studies conducted with adolescents have shown (Zavala-Berbena et al., 2008). In conclusion, EMOCINE is a novel and promising tool for the measurement of the perception and understanding branches of the EI ability in children. The test, employing film clips, is more realistic and motivating than others using static images or written descriptions.

## Data Availability

The datasets generated for this study are available on request to the corresponding author.

## Ethics Statement

The studies involving human participants were reviewed and approved by the Deontological Commission of the Faculty of Psychology of the Complutense University of Madrid (Ref. 2018/19-013). Written informed consent to participate in this study was provided by the participants’ legal guardian/next of kin.

## Author Contributions

All authors contributed equally to this work, except in the data analysis, in which only JA has performed.

## Conflict of Interest Statement

The authors declare that the research was conducted in the absence of any commercial or financial relationships that could be construed as a potential conflict of interest.

## Appendix I

LCA syntax in R to simulate and retrieve three latent classes and five items (Y1 to Y5). For simplicity, the five items are simulated equal with the following response probabilities for each class: class 1 probabilities of answer 1, 2, and 3 of 0.6, 0.3, and 0.1, respectively; class 2 probs. 0.2, 0.6, 0.2, and class 3 probs. 0.1,0.3, and 0.6.

# Example LCA

library(poLCA)

probs<- list(matrix(c(0.6,0.3,0.1, 0.2,0.6,0.2, 0.1,0.3,0.6),ncol=3,byrow=TRUE), # Y1

matrix(c(0.6,0.3,0.1, 0.2,0.6,0.2, 0.1,0.3,0.6),ncol=3,byrow=TRUE), # Y2

matrix(c(0.6,0.3,0.1, 0.2,0.6,0.2, 0.1,0.3,0.6),ncol=3,byrow=TRUE), # Y3

matrix(c(0.6,0.3,0.1, 0.2,0.6,0.2, 0.1,0.3,0.6),ncol=3,byrow=TRUE), # Y4

matrix(c(0.6,0.3,0.1, 0.2,0.6,0.2, 0.1,0.3,0.6),ncol=3,byrow=TRUE)) # Y5

# Sample size 1,000, with probabilities of each class 0.2, 0.3, and 0.5

simdat<- poLCA.simdata (N=1000, probs,P=c(0.3,0.4,0.3)) # syntax for simulation

mydata<-simdat$dat # Database with answers

f<- cbind(Y1,Y2,Y3,Y4,Y5)~1 # Recovery function is defined

lc3<-poLCA(f, data=mydata, nclass=3, na.rm=FALSE, nrep=30, maxiter=3000)

plot(lc3) # Graphic representation of the classes

lc3$predclass # The class to which each case belongs

## References

[B1] Barraca J. Fernández-González A. Sueiro M. (2009). *Test de sensibilidad a las Interacciones Sociales (TESIS): Una prueba objetiva para la medición de la inteligencia emocional.* Bizkaia: COHS.

[B2] Barrett L. F. Bliss-Moreau E. (2009). She’s emotional. He’s having a bad day: attributional explanations for emotion stereotypes. *Emotion* 9 649–658. 10.1037/a0016821 19803587

[B3] Brackett M. A. Katulak N. A. (2006). “Emotional intelligence in the classroom: skill-based training for teachers and students,” in *Improving Emotional Intelligence: A Practitioner’s Guide*, eds Ciarrochi J. Mayer J. D. (New York, NY: Psychology Press), 1–27.

[B4] Brackett M. A. Mayer J. D. Warner R. M. (2004). Emotional intelligence and its relation to everyday behaviour. *Person. Indiv. Dif.* 36 1387–1402. 10.1016/s0191-8869(03)00236-238

[B5] Brackett M. A. Rivers S. E. Shiffman S. Lernesr N. Salovey P. (2006). Relating emotional abilities to social functioning: a comparison of self-report and performance measures of emotional intelligence. *J. Pers. Soc. Psychol.* 91 780–795. 10.1037/0022-3514.91.4.780 17014299

[B6] Brown R. F. Schutte N. S. (2006). Direct and indirect relationships between emotional intelligence and subjective fatigue in university students. *J. Psychosom. Res.* 60 585–593. 10.1016/j.jpsychores.2006.05.001 16731233

[B7] Burns N. R. Bastian V. A. Nettelbeck T. (2007). “Emotional intelligence. more than personality and cognitive ability?,” in *The Science of Emotional Intelligence: Knowns and Unknowns*, eds Matthews G. Zeidner M. Roberts R. D. (Oxford: Oxford University Press), 167–196. 10.1093/acprof:oso/9780195181890.003.0007

[B8] Conte J. M. (2005). A review and critique of emotional intelligence measures. *J. Organ. Behav.* 26 433–440. 10.1002/job.319

[B9] Fernández-Berrocal P. Extremera N. (2016). Ability emotional intelligence, depression, and well-being. *Emot. Rev.* 8 311–315. 10.1177/1754073916650494

[B10] Fernández-Berrocal P. Ruiz-Aranda D. Salguero J. M. Palomera R. Extremera N. (2018). La relación del Test de Inteligencia Emocional de la Fundación Botín (TIEFBA) con el ajuste personal y escolar de adolescentes españoles. *Rev. Psicodidáctica* 23 1–8. 10.1016/j.psicod.2017.07.001

[B11] Fernández-Pinto I. Santamaría P. Sánchez-Sánchez F. Carrasco M. A. del Barrio V. (2015). *SENA. Sistema de Evaluación de Niños y Adolescentes. Manual técnico.* Madrid: TEA Ediciones.

[B12] Fischer A. H. Kret M. E. Broekens J. (2018). Gender differences in emotion perception and self-reported emotional intelligence: a test of the emotion sensitivity hypothesis. *PLoS One* 13:e0190712. 10.1371/journal.pone.0190712 29370198 PMC5784910

[B13] Fotopoulou E. Zafeiropoulos A. Alegre A. (2019). Improving social cohesion in educational environments based on a sociometric-oriented emotional intervention approach. *Educ. Sci.* 9:24 10.3390/educsci9010024

[B14] García-Sancho E. Salguero J. M. Fernández-Berrocal P. (2017). Ability emotional intelligence and its relation to aggression across time and age groups. *Scand. J. Psychol.* 58 43–51. 10.1111/sjop.12331 27678490

[B15] Goldenberg I. Matheson K. Mantler J. (2006). The assessment of emotional intelligence: a comparison of performance-based and self-report methodologies. *J. Pers. Assess.* 86 33–45. 10.1207/s15327752jpa8601_05 16436018

[B16] González J. García F. J. (2010). *SOCIOMET*. *Sociométrico.* Available at: http://web.teaediciones.com/sociomet.aspx (accessed April 4, 2019).

[B17] Linzer D. A. Lewis J. B. (2011). poLCA: an R package for polytomous variable latent class analysis. *J. Stat. Softw.* 42 1–29. 10.1111/jocn.13904 28557301

[B18] Martins A. Ramalho N. Morin E. (2010). A comprehensive meta-analysis of the relationship between emotional intelligence and health. *Person. Indiv. Differ.* 49 554–564. 10.1016/j.schres.2010 21111577

[B19] Mayer J. D. Caruso D. Salovey P. (1999). Emotional intelligence meets traditional standards for an intelligence. *Intelligence* 27 267–298. 10.1016/s0160-2896(99)00016-11 12934682

[B20] Mayer J. D. Caruso D. R. Salovey P. (2016). The ability model of emotional intelligence: principles and updates. *Emot. Rev.* 8 290–300. 10.1177/1754073916639667

[B21] Mayer J. D. Salovey P. (1997). “What is emotional intelligence?,” in *Emotional Development and Emotional Intelligence: Educational Implications*, eds Salovey P. Sluyter D. J. (New York, NY: Basic Books), 3–34.

[B22] Mayer J. D. Salovey P. Caruso D. R. (2002). *Mayer-Salovey-Caruso Emotional Intelligence Test (MSCEIT), version 2.0.* Toronto, ON: Multi-Health Systems.

[B23] Naghavi F. Redzuan M. (2011). The relationship between gender and emotional intelligence. *World Appl. Sci. J.* 15 555–561.

[B24] Omori M. Brackett M. A. Rivers S. Salovey P. (2006). *Emotional Intelligence, Selfesteem, and Maladaptive Behavior Among College Students.* Unpublished data, Yale University.

[B25] Rivers S. E. Brackett M. A. Reyes M. R. Mayer J. D. Caruso D. R. Salovey P. (2012). Measuring emotional intelligence in early adolescence with the MSCEIT-YV: psychometric properties and relationship with academic performance and psychosocial functioning. *J. Psychoeduc. Assess.* 30 344–366. 10.1177/0734282912449443

[B26] Salovey P. Mayer J. D. (1990). Emotional intelligence. *Imagin. Cogn. Pers.* 9 185–211. 10.2190/DUGG-P24E-52WK-6CDG

[B27] Sánchez-Sánchez F. Fernández-Pinto I. Santamaría P. Carrasco M. A. del Barrio V. (2016). SENA, Sistema de Evaluación de Niños y Adolescentes: proceso de desarrollo y evidencias de fiabilidad y validez. *Revista de Psicología Clínica con Niños y Adolescentes* 3 23–34.

[B28] Sastre S. Artola T. Alvarado J. M. (2018). Adolescents’ sensitivity in social interactions: an evaluation procedure using film clips. *Rev. Psicol. Soc.* 33 195–212. 10.1080/02134748.2017.1385244

[B29] Sharma D. (2017). Impact of age on emotional intelligence and its components. *Int. J. Res. Innov. Soc. Sci.* 1 13–20.

[B30] Śmieja M. Orzechowski J. Stolarski M. S. (2014). TIE: an ability test of emotional intelligence. *PLoS One* 9:e103484. 10.1371/journal.pone.0103484 25072656 PMC4114749

[B31] Van Rooy D. L. Alonso A. Viswesvaran C. (2005). Group differences in emotional intelligence test scores: theoretical and practical implications. *Pers. Individ. Dif.* 38 689–700. 10.1016/j.paid.2004.05.023

[B32] Vazire S. (2010). Who knows what about a person? The self-other knowledge asymmetry (SOKA) model. *J. Pers. Soc. Psychol.* 98 281–300. 10.1037/a0017908 20085401

[B33] Webb C. A. Schwab Z. J. Weber M. DelDonno S. Kipman M. Weiner M. R. (2013). Convergent and divergent validity of integrative versus mixed model measures of emotional intelligence. *Intelligence* 41 149–156. 10.1016/j.intell.2013.01.004

[B34] Zavala-Berbena M. A. Valadez-Sierra M. D. Vargas-Vivero M. C. (2008). Inteligencia Emocional y Habilidades Sociales en adolescents con alta aceptación social. *Rev. Electrón. Investig. Educ.* 6 319–338.

