# Corrigendum: Emotional Intelligence in Elementary School Children. EMOCINE, a Novel Assessment Test Based on the Interpretation of Cinema Scenes

**DOI:** 10.3389/fpsyg.2019.02031

**Published:** 2019-09-11

**Authors:** Santiago Sastre, Teresa Artola, Jesús M. Alvarado

**Affiliations:** ^1^University Center Villanueva, Complutense University of Madrid, Madrid, Spain; ^2^Department of Psychobiology & Behavioral Sciences Methods, Faculty of Psychology, Complutense University of Madrid, Madrid, Spain

**Keywords:** emotional intelligence, elementary children, test validity, films, emotional problems and disorders

In the original article, there was a mistake in the legend for **Figures 2, 3**, and **4** as published. The legends were not included in the article. The correct legends appear below.

“**Figure 2**. Distribution of latent classes regarding behavior in school/classroom. Different letters (a,b) indicate significant differences in sensitive responses”.

“**Figure 3**. Sociogram results regarding latent classes. Different letters (a,b) indicate significant differences in sensitive responses”.

“**Figure 4**. Emotional and behavioral problems from SENA questionnaire showing significant differences regarding latent classes”.

Additionally, there was a mistake for the department in affiliation 2. The correct department is “Department of Psychobiology & Behavioral Sciences Methods”.

The authors apologize for this error and state that this does not change the scientific conclusions of the article in any way. The original article has been updated.

