# Supplementary material for: Emotional Intelligence in Elementary School Children. EMOCINE, a Novel Assessment Test Based on the Interpretation of Cinema Scenes
Source: Front Psychol. 2019 Aug 14;10:1882. doi: 10.3389/fpsyg.2019.01882 (PMC6703194; doi:10.3389/fpsyg.2019.01882)
Supplement: Supplementary file 1 [file Data_Sheet_1.PDF]

Supplementary Table 1. Description of film clips and questions

Number	Movie	Scene description	Question and answers
1	Beauty and the beast (Disney, 1991)	Beauty and the beast are having dinner. The beast is trying to eat using a spoon, but it is difficult for him. Bella decides to eat without spoon.	Why does the beauty decide to eat without spoon?<br>A) Because she does not want the beast to be embarrassed for not being able to eat with spoon.<br>B) Because copy what the beast is doing.<br>C) Because the beast is sad, and the beauty would like to make him feel better.
2	Aladdin (Disney, 1992)	Jasmine has chosen Aladdin as a suitor. Her father assumes that Aladdin will be the following Sultan, being a sincere and honest man.	Why had Aladdin a guilty look?<br>A) Because he knew he is not being sincere.<br>B) Because he was afraid of being a Sultan.<br>C) Because he thought he is going to leave his family and friends.
3	Snow-white and the seven dwarfs (Disney, 1937)	Snow-white is kissing the forehead of each dwarf. Grumpy looks like he does not want to be kissed. However, when kissed, he liked so much that, even, stumbled.	Why is Grumpy so clumsy and stumble?<br>A) Because he is distracted, thinking on the snow-white kiss.<br>B) Because he is angry.<br>C) Because he is jealous.
4	The Jungle Book (Disney, 1967)	Mowgli and Bagheera are crying Baloo's death. Bagheera says nice words about him, and when leaving, Baloo opens the eyes and ask Bagheera for continuing the speech.	Why is Baloo crying when listening the nice words from Bagheera the panther?<br>A) Because he was saying good things about him.<br>B) Because the was saying sad things.<br>C) Because the panther was lying to Mowgli.
5	Sleeping Beauty (Disney, 1959)	Sleeping Beauty is dancing with her friends, the animals. Suddenly, Felipe the prince appears and attempts to dance with her. The beauty got surprised, and she refused to let him take her hand.	Why does the beauty not allow Felipe the prince taking her hand?<br>A) Because she is embarrassed.<br>B) Because she wanted to dance with the owl.<br>C) Because she avoids falling in love of him.
6	The Little Mermaid (Disney, 1989)	The King Triton is asking Sebastian if Ariel is in love. The crab thinks that Triton already knows Arial is with humans. Upon the insistency of Triton and in fear of being uncover, he tells the truth.	Why does Sebastian tell the truth to King Triton?<br>A) Because he thinks Triton already knows it.<br>B) Because he must be sincere.<br>C) Because he is in love of Ariel.

7	Mulan (Disney, 1998)	Mulan is sad, and her father get closer to console her. He, instead of talking directly about what is worrying to Mulan, talks about flowers, but referring to her creating a metaphor. He makes his daughter happy, who smiles.	Why does Mulan smile when her father talks about flowers?<br>A) Because he is referring to her.<br>B) Because she likes flowers.<br>C) Because she thinks she is beautiful.
8	Brave (Disney, 2012)	Merida is arguing with his mother because she does not want to be like her or behave in the way she likes. Merida get closer to a tapestry showing all her family and break it, separating her mother from the rest of them. Her mother throws the bow into the fire, although she regrets her decision quickly.	Why does Merida break the tapestry?<br>A) Because the mother does not let her behave like she is.<br>B) Because she is very angry with her mother.<br>C) Because she wants to be like her father.
9	Toy Story 3 (Pixar, 2010)	Andy gives all his toys to Bonnie. Suddenly, they realize that one of them, a cowboy, is still in the box. Andy is surprised to see it, he takes it, and looks it for a few seconds, not giving it to Bonnie.	Why is Andy not giving the cowboy to the girl?<br>A) Because he remembers when he was younger.<br>B) Because he likes too much that toy.<br>C) Because he thinks she could break it.
10	Wall-E (Pixar, 2008)	Wall-E is alone in the world. When arriving home, he starts watching a movie in which some persons are dancing. When a couple start singing, he stops doing what he was doing and pays special attention and, at the same time that in the movie, he joins the hands.	Why does the robot join the hands?<br>A) Because he would like to be accompanied by someone.<br>B) Because he is imitating what happens in the movie.<br>C) Because he cannot fall in love.
11	Rapunzel (Disney, 2010)	Rapunzel is trying to ask for her mother something very special; however, the mother is not paying attention, but continuing doing her tasks, and urges to her daughter to speak quickly. When Rapunzel dares to ask for it, the mother answers with surprises upon the request.	How is the mother behaving when Rapunzel talks?<br>A) With no interest, as if she did not care it.<br>B) In a strange and weird way.<br>C) Badly, because she is crazy.
12	Tarzan (Disney, 1999)	Jane is in the rainforest and is trying to run away from Tarzan. He is following her with interest and curiosity. Suddenly, he discovers that the glove has a hole, he takes it off, and joins his hands with hers, watching both hands with great attention.	Why does Tarzan take the glove to Jane?<br>A) Because he realizes that she is just like him.<br>B) Because it is broken.<br>C) Because he is in love with Jane.

13	Lady and the Tramp (Disney, 1995)	Lady is boarded in a kennel. Two dogs arrive and propose her, but not explicitly, marriage. Lady is, very kindly, rejecting them when another dog appears, and they turned their backs, angry.	What is the true proposition of the two dogs to Lady?<br>A) Get married.<br>B) Do not be sad.<br>C) They want to congratulate her for coming out from the kennel.
14	Dumbo (Disney, 1941)	Dumbo is getting closer to the caravan where his mother is locked up. She, when watching the son’s trunk, attempts to go with him, but, as locked up, she realizes angrily that she can`t go with him. For this reason, she uses her trunk for embracing him, rocking him, and singing him.	What does the Dumbo’s mother feel?<br>A) Joy, sad, and anger.<br>B) She feels completely happy for seeing Dumbo.<br>C) She is excited because she knows she will be released soon.
15	Pocahontas (Disney, 1995)	Pocahontas is following a weird boy that is in her lands. Suddenly, she is surprised because he is pointing at her with a shotgun. After watching a few seconds, the boy lowered the gun, and Pocahontas goes running away.	Why was Pocahontas getting closer to the boy?<br>A) Because she wanted to know who he was and what he was doing.<br>B) Because she wanted to see the waterfall.<br>C) Because they were, ultimately, in love.
