# Supplementary material for: Emotional Intelligence in Elementary School Children. EMOCINE, a Novel Assessment Test Based on the Interpretation of Cinema Scenes
Source: Front Psychol. 2019 Aug 14;10:1882. doi: 10.3389/fpsyg.2019.01882 (PMC6703194; doi:10.3389/fpsyg.2019.01882)
Supplement: Supplementary file 2 [file Data_Sheet_2.PDF]

Univariate Distributions of the Responses: 1 naïve, 2 over-interpretative and 3 sensitive

```
SCENE 1 Frequency Percentage Bar Chart
    1      52          8.0    □□□□□
    2     133         20.4    □□□□□□□□□□□□□
    3     468         71.7    □□□□□□□□□□□□□□□□□□□□□□□□□□□□□□□□□□□□□□□□□□□□□□□□

SCENE 2 Frequency Percentage Bar Chart
    1     190         29.1    □□□□□□□□□□□□□□□□□□□□□□□□□□□□□□□□□
    2     188         28.8    □□□□□□□□□□□□□□□□□□□□□□□□□□□□□□□□□
    3     275         42.1    □□□□□□□□□□□□□□□□□□□□□□□□□□□□□□□□□□□□□□□□□□□□□□□□

SCENE 3 Frequency Percentage Bar Chart
    1     108         16.5    □□□□□□□□□□
    2      57          8.7    □□□□□
    3     488         74.7    □□□□□□□□□□□□□□□□□□□□□□□□□□□□□□□□□□□□□□□□□□□□□□□□

SCENE 4 Frequency Percentage Bar Chart
    1      92         14.1    □□□□□□□□
    2      32          4.9    □□□
    3     529         81.0    □□□□□□□□□□□□□□□□□□□□□□□□□□□□□□□□□□□□□□□□□□□□□□□□

SCENE 5 Frequency Percentage Bar Chart
    1     163         25.0    □□□□□□□□□□□□□□□□□□□□□□
    2     139         21.3    □□□□□□□□□□□□□□□□□□□
    3     351         53.8    □□□□□□□□□□□□□□□□□□□□□□□□□□□□□□□□□□□□□□□□□□□□□□□□

SCENE 6 Frequency Percentage Bar Chart
    1     396         60.6    □□□□□□□□□□□□□□□□□□□□□□□□□□□□□□□□□□□□□□□□□□□□□□□□
    2      76         11.6    □□□□□□□□□
    3     181         27.7    □□□□□□□□□□□□□□□□□□□□□

SCENE 7 Frequency Percentage Bar Chart
    1      76         11.6    □□□□□□□
    2      59          9.0    □□□□□
    3     518         79.3    □□□□□□□□□□□□□□□□□□□□□□□□□□□□□□□□□□□□□□□□□□□□□□□□

SCENE 8 Frequency Percentage Bar Chart
    1     193         29.6    □□□□□□□□□□□□□□□□□□□□□□
    2      39          6.0    □□□□
    3     421         64.5    □□□□□□□□□□□□□□□□□□□□□□□□□□□□□□□□□□□□□□□□□□□□□□□□

SCENE 9 Frequency Percentage Bar Chart
    1     137         21.0    □□□□□□□□□□□□□
    2      29          4.4    □□□
    3     487         74.6    □□□□□□□□□□□□□□□□□□□□□□□□□□□□□□□□□□□□□□□□□□□□□□□□

SCENE 10 Frequency Percentage Bar Chart
    1      70         10.7    □□□□□□□
    2      93         14.2    □□□□□□□□□
    3     490         75.0    □□□□□□□□□□□□□□□□□□□□□□□□□□□□□□□□□□□□□□□□□□□□□□□□

SCENE 11 Frequency Percentage Bar Chart
    1     197         30.2    □□□□□□□□□□□□□□□□□□□□□□□□□□
    2      93         14.2    □□□□□□□□□□□□
    3     363         55.6    □□□□□□□□□□□□□□□□□□□□□□□□□□□□□□□□□□□□□□□□□□□□□□□□

SCENE 12 Frequency Percentage Bar Chart
    1     112         17.2    □□□□□□□□□□□□□□□□
    2     207         31.7    □□□□□□□□□□□□□□□□□□□□□□□□□□□□□
    3     334         51.1    □□□□□□□□□□□□□□□□□□□□□□□□□□□□□□□□□□□□□□□□□□□□□□□□

SCENE 13 Frequency Percentage Bar Chart
    1     425         65.1    □□□□□□□□□□□□□□□□□□□□□□□□□□□□□□□□□□□□□□□□□□□□□□□□
    2      66         10.1    □□□□□□□
    3     162         24.8    □□□□□□□□□□□□□□□□□□
```

SCENE 14	Frequency	Percentage	Bar Chart
1	576	88.2	□□□
2	34	5.2	□□□
3	43	6.6	□□□□

SCENE 15	Frequency	Percentage	Bar Chart
1	23	3.5	□□□
2	340	52.1	□□□
3	290	44.4	□□
